# Research on unit circuits based on cathode modulated vacuum/air channel electron tube

**DOI:** 10.1038/s41378-026-01234-z

**Published:** 2026-04-20

**Authors:** Wenjing Ying, Ziyi Lai, Haitao Xu, Minglv Wang, Qiufeng Ye, Zebo Fang, Jingquan Liu, Yuelin Wang

**Affiliations:** 1 National Key Laboratory of Advanced Micro and Nano Manufacture Technology, Shanghai Jiao Tong University, Shanghai, China; 2 DCI Joint Team, Collaborative Innovation Center of IFSA, School of Integrated Circuits (School of Information Science and Electronic Engineering), Shanghai Jiao Tong University, Shanghai, China; 3 Zhejiang Engineering Research Center of MEMS, Shaoxing University, Shaoxing, China

**Keywords:** Electrical and electronic engineering, Electronic devices

## Abstract

Over the past decade, the reported planar vacuum electron tubes (VETs) have not yet succeeded in resolving the gate leakage issue due to inherent limitations of their working principles, preventing their circuit integration thus far. This paper proposes a cathode modulated vacuum/air channel electron tube (CMVET), which completely solves the long-standing gate leakage problem. Furthermore, the developed CMVET has been integrated into fundamental circuit blocks and successfully applied in common-source amplifiers, differential amplifiers, cascode amplifiers, as well as NOR and NAND gates, thereby demonstrating its functionality and laying a foundation for further research on monolithic electron tube integrated circuits.

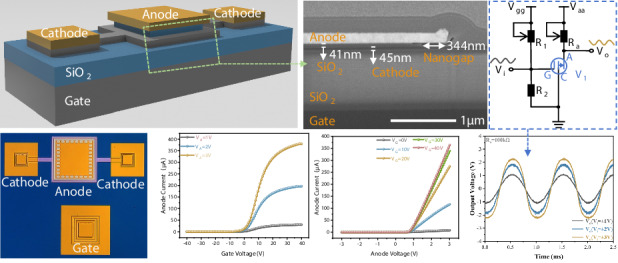

## Introduction

Transistors, as the foundation of integrated circuits (ICs), have undergone continuous miniaturization and have achieved higher speed over the past half-century^1^. As the feature sizes of transistors enter the nanometer scale, their speed is approaching the saturation velocity of carriers in solids, which is on the order of 10^7 ^cm/s, due to scattering and other issues encountered by carriers in motion^2^. Improving this performance is extremely challenging. By contrast, the theoretical speed of electrons in vacuum tubes can reach the speed of light (3×10^10 ^cm/s), which is approximately three orders of magnitude higher than that of transistors^3^. Consequently, if vacuum tubes could be employed as the fundamental building block for vacuum tube integrated circuits, their performance could be significantly enhanced.

Over a decade, numerous research groups around the world have investigated the fabrication of planar vacuum electron tubes (VETs) using microelectronics technology for their application in integrated circuits^1–17^. The reported device structures of VETs can generally be categorized into four primary structures: the gate on top^10^, the gate between the anode and cathode^7,8,11,12,15^, the gate at the bottom^1–6^, and the gate on the same plane as the anode and cathode^9,13,14^. All of the aforementioned VET structures operate by emitting electrons from the cathode and modulating the gate voltage to change the number of electrons reaching the anode, thereby regulating the anode current via the gate. This brings about a fatal issue: if all electrons from the cathode reach the anode, it is merely a diode; if the gate controls the electrons so that not all reach the anode, then the electrons can only go to the gate, resulting in gate leakage current. Owing to this fundamental flaw in the working principle, reported VETs have not yet been integrated into functional circuits.

To overcome this challenge, we propose a VET with gate-controlled cathode electron concentration (CMVET). By controlling the electron concentration at the cathode via the gate voltage, the purpose of controlling the cathode field emission current is achieved. All electrons emitted from the cathode reach the anode, meaning that the cathode current equals the anode current, which effectively eliminates the gate leakage current issue. The CMVET we have developed has been constructed into typical unit circuits and has been successfully applied in unit circuits such as common-source amplifiers, differential amplifiers, cascode amplifiers, as well as NOR and NAND gates. This paves the way for further research into monolithic vacuum tube integrated circuits.

## Results and discussion

### Principle and electrical characteristic of the CMVET

Equation (1) shows the equation of field emission current density^18^, where J is the field emission current density, N(W, T) is the electron density with the kinetic energy W in the tunneling direction at temperature T(K), and D(F, W, T) represents the transmission coefficient or tunneling probability.

As inferred from equation (1), the field emission current density is related to the electron concentration at the cathode and the electron penetration probability. The field emission current density increases with the increase in electron concentration (or: the field emission current density is positively correlated with electron concentration). Unlike in metals where the electron concentration is essentially a constant, the electron concentration in silicon is related to the doping concentration and modulation voltage. Therefore, when silicon is used as a field emission cathode, by regulating the electron concentration of the silicon cathode through gate voltage, it is possible to control the field emission current from the cathode, thereby realizing the function of a VET.

1 $${\rm{J}}\left({\rm{F}},{\rm{T}}\right)={\rm{e}}{\int }_{0}^{\infty }{\rm{N}}\left({\rm{W}},{\rm{T}}\right){\rm{D}}\left({\rm{F}},{\rm{W}},{\rm{T}}\right){\rm{dW}}$$

**Fig. 1:**
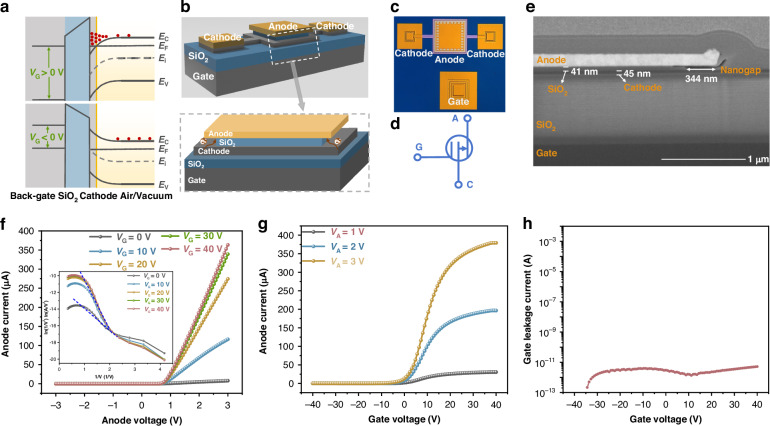
Working principle, device structure, chip photo, device symbols and device characteristics of CMVET. **a** The band schematic diagram of the device’s working principle. **b** Schematic of the CMVET device section. **c** Chip photo. **d** Device symbol. **e** SEM diagram of the device. **f** Output characteristic (the inserted figure is the F-N plot). **g** Transfer characteristic. **h** Gate leakage current

The working principle of this device can be explained by the energy band diagram in Fig. 1a. When V_G_ is positive, electrons in the cathode are attracted to the cathode-gate surface and accumulate, causing the energy band to bend downward from the cathode-gate interface. Conversely, when V_G_ is negative, electrons are repelled into the interior of semiconductor and an electron depletion region gradually forms, resulting in an upward band bending at the cathode-gate boundary. Provided the cathode is sufficiently thin (45 nm in this work), this band bending propagates to the cathode-air surface. Consequently, the electron concentration at the field-emission surface of cathode is modulated and the electron-emission characteristic of cathode is modulated as well.

The CMVET structure is adapted from our previously published diode in 2021^19,20^, as illustrated in Fig. 1b. The starting material for the chip is an SOI silicon wafer, with an n-type silicon substrate serving as the back gate. The device is fabricated using IC-compatible steps including thermal oxidation, chemical-vapor deposition (CVD), dry etching and magnetron sputtering. The nanogap is ultimately defined by etching the sacrificial layer between anode and cathode with buffered oxide etchant (BOE). Silicon dioxide on the substrate acts as the gate oxide layer, and the n-type silicon on top of the gate oxide layer serves as the cathode. The vacuum/air channel above the cathode functions as the field emission current channel, with the top layer of gold acting as the anode. The specific working principle is as follows: when a positive voltage is applied to the anode, due to the vacuum/air layer channel between the anode and cathode being only tens of nanometers, field emission occurs at the cathode under the influence of the anode voltage, forming a field emission current. At this point, if a gate voltage is applied between the back gate and the cathode, the electron concentration in the cathode will increase with the increase in gate voltage, thereby increasing the field emission current as the gate voltage increases. This achieves the purpose of regulating the field emission current between the anode and cathode, realizing the function of an electron tube. The device has a gate oxide layer between the cathode and the gate, ensuring minimal gate leakage current.

The device is fabricated using conventional integrated circuit processes such as oxidation, ion implantation, etching, and deposition, which are compatible with IC fabrication techniques. The chip photograph, circuit symbol and SEM diagram are shown in Fig. 1c–e. The transfer characteristics, output characteristics, and gate leakage current of the device are shown in Fig. 1f–h. The device exhibits a switch current ratio of approximately 10^4^, a transconductance of about 23 μS (with V_A_ = 3 V and V_G_ = 10 V), and the gate leakage current is less than 10^−11 ^A. For a direct performance comparison, Table 1 summarizes key metrics of our CMVET against other reported VETs, where I_A_ denotes anode current, I_G_ gate leakage current, and g_m_ transconductance. It is noteworthy that while references^1,8^ also report low gate leakage due to an insulating layer, their operating principle can lead to charge trapping within the insulator over time, giving rise to a long-term electrostatic charge accumulation effect.

**Table 1:** Comparison of electrical properties of different VETs

Ref	Nanogap	I_A_	I_G_	g_m_
^21^	200 nm	11.4 μA@V_A_ = 20 V, V_G_ = 20 V	10^−8^ A	/
^8^	50 nm	3 μA@V_A_ = 2 V, V_G_ = 5 V	10^−12^ A	34 μS
^22^	50 nm	8000 μA@V_A_ = 0.7 V, V_G_ = −0.8 V	10^−5^ A	/
^1^	150 nm	40 μA@V_A_ = 20 V, V_G_ = 8 V	10^−11^ A	0.2 μS
^11^	367 nm	−0.37 μA@V_A_ = −150 V, V_G_ = −30 V, 150 °C	10^−9^ A	24.5 nS
**This work**	**41** **nm**	**370 μA@V**_**A**_ = **3** **V, V**_**G**_ = **40** **V**	**10**^**−12**^ **A**	**23 μS**

### Unit circuit building and testing

**Fig. 2:**
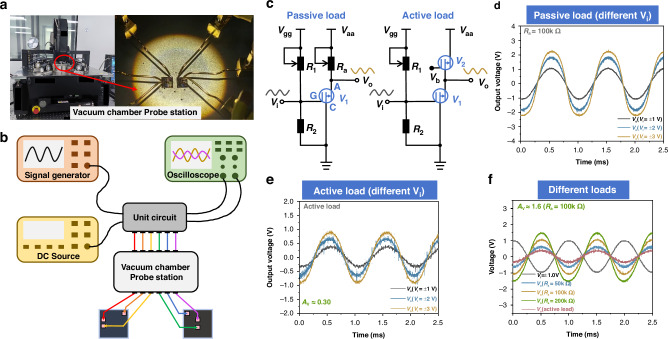
Unit circuit test system and common source amplifier circuit based on CMVET. **a** Sample setup inside probe station. **b** Schematic diagram of the complete test system. **c** Schematic diagram of circuit. **d** Output of the passive load under input signals of different amplitudes. **e** Output of an active load under input signals of different amplitudes. **f** Output under different loads

Currently, the reported VETs have not been integrated into circuits due to inherent limitations in their working principles. The CMVET we have developed has minimal gate leakage, and whether it can function as a transistor in circuits needs to be verified by applying the CMVET to specific circuits. To this end, we constructed basic circuit blocks such as common-source amplifiers, differential amplifiers, cascode amplifiers, NOR, and NAND gates using the CMVET. We then correlated the performance of these CMVET-based circuits with key device characteristics. It is noteworthy that, as can be seen from the device output characteristics in Fig. 1f–h, the CMVET is a non-saturating device, whose field emission current always increases with the increase of voltage between the anode and cathode, unlike MOSFETs that exhibit saturation when the source-drain voltage increases to a certain value. This characteristic makes the application of CMVET in circuits distinctly different from that of MOSFETs, which is an issue that needs special consideration when building circuits with CMVET. Figure 2a and b showcase the experimental setup for testing these CMVET-based circuit blocks at room temperature and atmospheric pressure, representing a hybrid integration of on-prober devices with external electrical instrumentation and interconnects.

First, we constructed a common-source amplifier circuit based on the CMVET, as shown in Fig. 2c. By varying the amplitude of the input signal (V_i_), we investigated the amplification characteristics of the circuit. For the passive load amplifier circuit, the operating point was set with a gate voltage (V_G_) of 10 V and an anode voltage (V_A_) of 4 V. The relationship between the output signal (V_o_) and the input signal (V_i_) is shown in Fig. 2d. The output signal increases with the increase of the input signal, indicating that the CMVET has realized the function of an amplification circuit. For the active load amplifier circuit, we replaced the load resistor with another CMVET. The relationship between the tested output signal (V_o_) and the input signal (V_i_) is shown in Fig. 2e. Similarly, the output signal increases with the increase of the input signal, but the gain is smaller than that of the passive load. We also explored the relationship between the performance of the common-source amplifier circuit and the load resistance by changing the load resistor R_a_. By adjusting V_aa_ to keep the anode voltage (V_A_) constant at 4 V, we changed the load resistance without altering the operating point of the CMVET. The relationship between the tested output signal (V_o_) and the input signal (V_i_) is shown in Fig. 2f. The results indicate that the circuit gain increases with the increase of the load resistance, peaking at approximately 1.6 when the load is 200 kΩ. Among them, the gain is the smallest when using an active load, which is due to the fact that the CMVET is a non-saturating device with a relatively low output impedance. The actual equivalent resistance of the load electron tube V_2_ is approximately 30 kΩ, which is the smallest load resistance, hence its gain is also the smallest, only 0.3. Therefore, reducing the non-saturating characteristics of the CMVET and how to avoid the impact of non-saturating characteristics in circuit applications is very important.

**Fig. 3:**
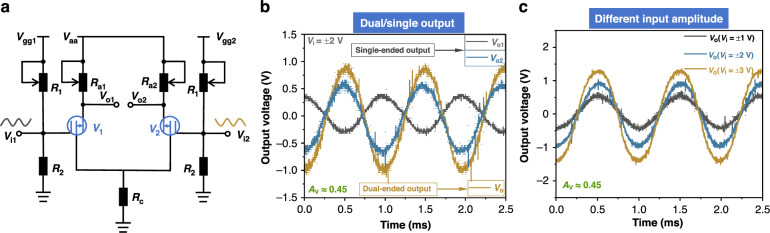
Schematic diagram of differential amplifier circuit and test results. **a** Schematic diagram of circuit. **b** Differential mode input single/double-ended output. **c** Double-ended output under different-mode inputs of different amplitudes

The differential amplifier circuit we constructed is shown in Fig. 3a. Using this circuit, we explored the single-ended/double-ended output scenarios when inputting a differential signal. The gate voltage was set to 10 V, and V_aa_ was set to 10 V. With a differential input signal applied to both ends of the circuit, we tested the single-ended output and double-ended output at both ports, and the test results are shown in Fig. 3b. The results indicate that for the differential input signal, the single-ended output signals at the corresponding two ports are phase-shifted by 180°, while the double-ended output is the difference between the single-ended output signals at the two ports, and the gain is 0.45. We also varied the amplitude of the differential input signal and tested the changes at the output end, with the results shown in Fig. 3c. The output signal increased with the increase of the input signal with a gain of approximately 0.45, demonstrating that the CMVET has achieved the function of a differential amplifier.

**Fig. 4:**
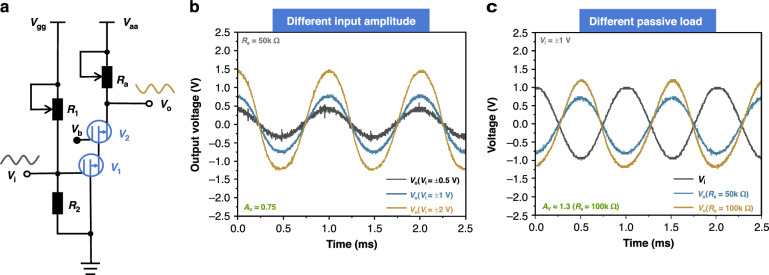
Schematic diagram of cascode circuit and test results. **a** Schematic diagram of circuit. **b** Outputs at different input signal amplitudes. **c** Output under different loads

Based on the CMVET, we constructed a cascode amplifier circuit and investigated its characteristics. The circuit is depicted in Fig. 4a, with V_1_ CMVET gate voltage set to 13 V, V_2_ CMVET gate voltage set to 12 V, V_1_ CMVET anode voltage set to 4 V, and V_2_ CMVET anode voltage set to 2 V. When the load resistance R_a_ is 50 kΩ, the relationship between the circuit’s output signal and input signal is shown in Fig. 4b. The output signal increases with the increase of the input signal with a gain of approximately 0.75, indicating that the CMVET has fulfilled the function of the cascode amplifier circuit. By altering the load resistance R_a_, we also studied the relationship between circuit gain and load resistance. The test results are presented in Fig. 4c and the gain peaks at approximately 1.3 when the load is 100 kΩ, which demonstrate that the circuit gain increases as the load resistance increases.

The results presented above indicate that the CMVET we have developed can be applied to analog unit circuits. To explore the potential application of CMVET in digital circuits, we have also constructed two typical logic circuits, namely a NAND gate and a NOR gate, to investigate the performance of CMVET in logical circuits.

**Fig. 5:**
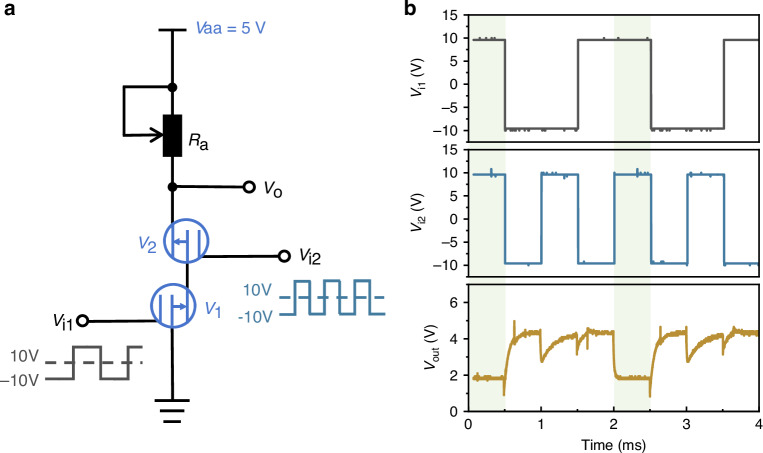
Schematic diagram of NAND gate circuit and test results. **a** Schematic diagram of circuit. **b** Results of the NAND gate test

The constructed NAND gate circuit is shown in Fig. 5a. The input signal V_i1_ has an amplitude of ±10 V and a frequency of 500 Hz, while V_i2_ has an amplitude of ±10 V and a frequency of 1000 Hz. The test results are depicted in Fig. 5b. When both input signals to V_1_ and V_2_ are at a high level, both CMVETs conduct, resulting in a low output voltage (V_o_) of approximately 1.9 V. When one of the input signals to either V_1_ or V_2_ is at a low level, the device with the low input signal becomes non-conducting, and the output V_o_ is at a high level, approximately 4.5 V. The results demonstrate that the CMVET can achieve the functionality of a NAND gate circuit.

**Fig. 6:**
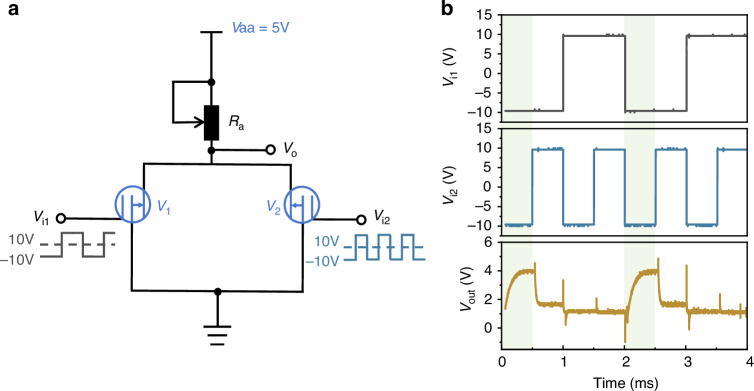
Schematic diagram of the NOR gate circuit and test results. **a** Schematic circuit. **b** Results of NOR gate test

The constructed NOR gate circuit is illustrated in Fig. 6a. The input signal V_i1_ has an amplitude of ±10 V with a frequency of 500 Hz, while V_i2_ has an amplitude of ±10 V with a frequency of 1000 Hz. The testing results are presented in Fig. 6b. When both input signals to V_1_ and V_2_ are at a low level, both CMVETs are cut off, resulting in a high output voltage (V_o_) of approximately 4 V. When one of the input signals to either V_1_ or V_2_ is at a high level, the device with the high input signal conducts, leading to a low output voltage (V_o_) of approximately 1.1 V. The results indicate that the CMVET is capable of implementing the functionality of a NOR gate circuit.

## Conclusion

We propose a cathode modulated vacuum/air channel electron tube (CMVET), which completely resolves the long-standing issue of gate leakage current. Based on our innovative CMVET, we have constructed common-source amplifiers, differential amplifiers, cascode amplifiers, NAND gates, and NOR gates, which are commonly used unit circuits. Experimental results confirm that the CMVET can perform the corresponding circuit functions in the aforementioned unit circuits, demonstrating its capability to act as a functional transistor-like device for signal processing. To our knowledge, this represents the first successful implementation of a vacuum/air-channel electron tube in integrated circuit blocks. Undoubtedly, the device performance requires further optimization. For instance, similar to electron tubes, the field emission current of the CMVET always increases monotonically with the increasing anode voltage, making it a non-saturating device. Unlike MOSFETs, the current does not saturate when the source-drain voltage increases to a certain value. Therefore, reducing the non-saturating characteristics of the CMVET and avoiding the impact of non-saturating characteristics in circuit applications are of great importance. However, it cannot be denied that this work provides a solid foundation for further research on monolithic electron tube integrated circuits.

## Data Availability

The data that support the findings of this study are available from the corresponding author upon reasonable request.
